# Längeres Arbeitslosengeld in der Krise? Covid-19 und die angemessene maximale Bezugsdauer

**DOI:** 10.1007/s11577-021-00806-3

**Published:** 2021-12-06

**Authors:** Christopher Osiander, Monika Senghaas, Gesine Stephan, Olaf Struck

**Affiliations:** 1 Institut für Arbeitsmarkt- und Berufsforschung (IAB), Bundesagentur für Arbeit (BA), Regensburger Straße 104, 90478 Nürnberg, Deutschland; 2 FB Wirtschafts- und Sozialwissenschaften, Friedrich-Alexander-Universität Erlangen-Nürnberg, Postfach 3931, 90020 Nürnberg, Deutschland; 3 SOWI-Fakultät, Prof. für Arbeitswissenschaft, Universität Bamberg, Feldkirchenstraße 21, 96045 Bamberg, Deutschland

**Keywords:** Sozialversicherung, Covid-19, Gerechtigkeit, Faktorielles Survey, Vignetten, Social security, Covid-19, Justice, Factorial survey, Vignettes

## Abstract

Dieser Beitrag untersucht die Frage, welche Bezugsdauern des Arbeitslosengeldes für welche Personengruppen als angemessen betrachtet werden und ob sich diese Einschätzungen während der Covid-19-Pandemie verändert haben. Längere Bezugsdauern können die Einkommenssituation der Betroffenen stabilisieren und die Suche nach einer qualifikationsadäquaten oder besser entlohnten Stelle unterstützen. Längere Zahlungen mindern aber auch den Druck zur Arbeitsaufnahme, wodurch die Dauer der Arbeitslosigkeit zunimmt. Verändern Menschen Abwägungen zwischen individuellem Bedarf, Leistung und gesellschaftlicher Effizienz in der gesundheitlichen und ökonomischen Krise? Wir untersuchen mithilfe von Daten aus Onlinebefragungen im November 2019 und in der Krise im Mai 2020, welche Bezugsdauern (zumeist) Erwerbstätige für angemessen erachten. Dazu wurden den Teilnehmenden Vignetten mit Beschreibungen hypothetischer Arbeitsloser vorgelegt, deren Charakteristika zufällig variieren. Die Ergebnisse zeigen, dass dieselben Befragten vor und während der Krise sehr ähnliche Bezugsdauern für angemessen halten. Dabei beziehen sie bei der Bemessung der Bezugsdauer für Arbeitslose neben Beitragsprinzipien auch Kriterien der Bedürftigkeit mit ein. So beeinflussen Merkmale wie etwa das Alter der Arbeitslosen, ihr eigenes (Nicht‑)Verschulden, ihre Lebensleistungen oder die Länge ihrer Beitragszahlungen das Urteil, welche Dauer des Leistungsbezugs als angemessen angesehen wird.

## Abstract (en)

This article deals with the question of which unemployment benefit durations are considered fair for which groups. In addition, it examines the extent to which individuals consider longer unemployment insurance benefit durations to be appropriate in times of economic crisis, such as the current situation during the Covid-19 pandemic. Longer reference periods can stabilize the income situation of benefit recipients and can provide time to search for an adequate job and thus increase matching quality. However, they also initially reduce the pressure to look for a job, and they lengthen the period of unemployment in the longer term. Using survey data from two online surveys done in November 2019 and during the crisis in May 2020, we examine which unemployment benefit durations employees consider appropriate. For this purpose, we presented vignettes to the survey participants describing hypothetical unemployed people whose characteristics varied randomly. The results show that the same respondents considered similar reference periods to be appropriate at both dates. In addition, the respondents took into account criteria of contribution as well as neediness when assessing the appropriate duration of benefits for the unemployed. Characteristics such as the age of the unemployed and any existing culpability, life benefits, or contribution periods influenced the duration of the benefit receipt that respondents judged to be appropriate.

## Einführung

Wohlfahrtsstaatlichen Institutionen und ihren Systemen und Regeln der (Um‑)Verteilung liegen spezifische Vorstellungen über angemessene Unterstützungsleistungen – etwa im Falle von Nichterwerbstätigkeit – zugrunde. Diese können sehr gering sein, sodass soziale Sicherheit insbesondere über Eigenverantwortung etwa auf dem Arbeitsmarkt oder durch die Familien gewährt wird. Oder staatliche Sozialpolitik bietet Leistungen an, die dann wiederum mehr oder minder an der Höhe der Bedürfnisse und/oder an Eigenleistungen orientiert sind. Hohe Bedeutung haben hierbei Gerechtigkeitseinstellungen, die sich auf Verteilungen zwischen Leistungen und Gegenleistungen oder auch Bedarfe richten können (Leisering 2004). Hier dokumentieren viele Autoren seit mehr als zehn Jahren Akzentverschiebungen in Richtung einer investiven Sozialpolitik, die auch gesellschaftliche Erträge erzielen soll (Sachweh et al. 2009, S. 612; Vobruba 1996). Für die Legitimität des Wohlfahrtstaates ist es von Bedeutung, dass Menschen sozialpolitische Maßnahmen und Regelungen akzeptieren und als gerecht empfinden (Sachweh et al. 2009, S. 618; Rothstein 1998; Roosma et al. 2013).

Dieser Beitrag untersucht die Einstellungen der Erwerbsbevölkerung zu maximalen Bezugsdauern des Arbeitslosengeldes. Ein besonderer Fokus dieser Studie liegt darauf, ob sich diese Einstellungen infolge der Covid-19-Pandemie verändert haben. Die maximalen Bezugsdauern sind in Deutschland immer wieder Gegenstand politischer Diskussionen und wurden in der Vergangenheit, anders als beispielsweise die Höhe des Arbeitslosengeldes, wiederholt neu ausgestaltet. Eine temporäre Verlängerung der Bezugsdauer, wie sie in der Covid-19-Pandemie erfolgt ist, ist allerdings für Deutschland ein Novum. In den USA sind solche „extended benefits“ in Krisenzeiten bereits seit Langem im Einsatz (z. B. Howell und Azizoglu 2011).

Bei der Festlegung der maximalen Bezugsdauern müssen die politischen Entscheidungsträger verschiedene Argumente abwägen, die jeweils wiederum Verteilungen von Gerechtigkeitsprinzipien widerspiegeln. Einerseits soll Arbeitslosengeld aus der von sozialversicherungspflichtig Beschäftigten und Arbeitgebern getragenen Arbeitslosenversicherung es ermöglichen, dass Menschen im Falle von Arbeitslosigkeit nicht sofort in finanzielle Not geraten (etwa aufgrund von fortlaufenden Ausgaben wie Miete oder für Kredite). Auch kann es für bessere Passungen zwischen Arbeitsuchenden und offenen Stellen oder den Erhalt von Qualifikationen volkswirtschaftlich sinnvoll sein, dass arbeitslos gewordene Personen nicht unmittelbar jede, auch eine nicht den eigenen Qualifikationen entsprechende Arbeit annehmen müssen. Andererseits sollen Leistungen des Sozialstaates keine Anreize für eine verzögerte oder verringerte Suche nach einer neuen Stelle bieten. Wenn mit der maximalen Bezugsdauer die Arbeitslosigkeitsdauer steigt, führt dies nicht nur zu (zusätzlichen) Ausgaben für die Arbeitslosenversicherung, sondern kann auch mit (zusätzlichen) Qualifikationsentwertungen einhergehen. Zudem können Arbeitgeber längere Arbeitslosigkeitsdauern von Bewerberinnen und Bewerbern als negatives Signal interpretieren. Für Deutschland weist eine Anzahl von Studien kausal nach, dass die Dauer der Arbeitslosigkeit mit der maximalen Bezugsdauer des Arbeitslosengeldes zunimmt (z. B. Riphahn und Schrader 2020; Schmieder et al. 2012). Hingegen ist die Literatur nicht eindeutig, was die Effekte der maximalen Bezugsdauer auf die Beschäftigungsqualität betrifft (z. B. Caliendo et al. 2013; Schmieder et al. 2016).

Es gibt einige Argumente dafür, in Krisenzeiten länger Arbeitslosengeld zu zahlen als in wirtschaftlichen Boomphasen (Dietz et al. 2009). Längere Bezugsdauern erhöhen die Sicherheit für Personen, die in Krisen besondere Schwierigkeiten haben, eine neue Stelle zu finden. Personen, die nach Auslaufen des Arbeitslosengeldes keinen Anspruch auf Leistungen der Grundsicherung haben, brauchen Ersparnisse länger nicht anzugreifen. Darüber hinaus kann eine längere Arbeitslosenunterstützung in der Krise als Stabilisator der Konsumnachfrage dienen.

Solche für politische Entscheidungen bedeutsamen und genauen Abwägungen zwischen aktueller Bedürftigkeit und individuellem Leistungserhalt, dem Setzen von Leistungsanreizen und der Wahrung gesellschaftlicher oder wirtschaftlicher Produktivität werden Menschen vermutlich nicht immer anstellen, wenn sie ad hoc Entscheidungen über die maximale Bezugsdauer des Arbeitslosengeldes treffen sollen, die sie als gerechtfertigt empfinden.

Allerdings zeigen Befunde zur als angemessen empfundenen Höhe des Arbeitslosengeldes II, dass Menschen bei ihren Einschätzungen etwa die persönlichen Merkmale der potenziellen Empfängerinnen und Empfänger in Betracht ziehen. Eine besonders wichtige Rolle für die zugesprochene Höhe des Arbeitslosengeldes spielt dabei, ob Kinder im Haushalt vorhanden sind (Buss 2019; Hörstermann und Andreß 2015). Für die Arbeitslosenversicherung wurde gezeigt, dass Merkmale der Leistungsbeziehenden, wie beispielsweise das Alter, Urteile von Befragten über die Zumutbarkeit von Stellenangeboten beeinflussen (Osiander und Senghaas 2020). Unseres Wissens liegen bisher keine empirischen Befunde dazu vor, welche Bezugsdauern des Arbeitslosengeldes in Deutschland als angemessen angesehen werden. Unbekannt ist zudem, ob sich Menschen an Gerechtigkeitsmerkmalen orientieren, die Bedürftigkeit oder individuelle Leistungen (Adams 1965; Kluegel et al. 1999; Gilliland 1993; Liebig 1997; van Oorschot 2000; Wegener 1992) widerspiegeln, und inwieweit sich in Entscheidungen auch produktivistische Gerechtigkeitsvorstellungen (Leisering 1999, 2004; Vobruba 1996) niederschlagen. Unbekannt ist zudem, ob Menschen in Krisenzeiten ihre Urteile über Bezugsdauern verändern und ob es zu Veränderungen von Gerechtigkeitsvorstellungen kommt.

Wir untersuchen daher, welche maximale Bezugsdauer des Arbeitslosengeldes (überwiegend) beschäftigte Personen hypothetischen Arbeitslosen vor der Covid-19-Pandemie Ende 2019 und während der ersten Hochphase der Pandemie und des Lockdowns im Mai 2020 zusprechen würden. In Abschn. 2 gehen wir kurz auf den institutionellen Rahmen unseres Untersuchungsgegenstands ein. In Abschn. 3 wird der theoretische und empirische Forschungsstand skizziert und in Hypothesen überführt. Abschnitt 4 erläutert Datensatz und Methode. In einer Panel-Onlinebefragung variieren wir in Vignetten Merkmale hypothetischer Arbeitsloser, die teilweise auch die Gesetzgebung aktuell heranzieht, wie das Alter und die Versicherungsdauer, aber auch den Haushaltskontext. Hierdurch wird deutlich, auf welchen Gerechtigkeitsprinzipien Bewertungen basieren. Die Ergebnisse der Analysen werden im Abschn. 5 vorgestellt und diskutiert. Ein Fazit schließt den Aufsatz ab.

## Institutioneller Rahmen von Bezugsdauern des Arbeitslosengeldes

Nach dem Sozialgesetzbuch III (SGB III) haben in Deutschland Personen bei Arbeitslosigkeit Anspruch auf Arbeitslosengeld, wenn sie sich bei der Agentur für Arbeit arbeitslos melden und die sogenannte Anwartschaftszeit erfüllen (§ 137 und § 142 SGB III). Letztere hat eine Person erfüllt, die in der sogenannten Rahmenfrist mindestens zwölf Monate in einem Versicherungspflichtverhältnis gestanden hat (§ 143 und § 24 SGB III). Die Rahmenfrist wurde Anfang 2020 von 24 Monaten auf 30 Monate erweitert. Bei überwiegend kurzen Beschäftigungsverhältnissen sieht das Gesetz unter bestimmten Bedingungen eine verkürzte Anwartschaftszeit von sechs Monaten vor; diese Regel nutzen aber weniger als 1000 Personen pro Jahr (Werner et al. 2012).

Wird ein Anspruch auf Arbeitslosengeld festgestellt, so hängt die maximale Bezugsdauer a) von der Dauer des vorangegangenen Beschäftigungsverhältnisses in der um 30 Monate erweiterten Rahmenfrist und b) vom Lebensalter ab. Tabelle 1 gibt einen Überblick über die gesetzlichen Regelungen zur maximalen Bezugsdauer. Diese gelten seit dem 1. Januar 2008 im Grundsatz unverändert. Im Sozialschutzpaket II, das der Bekämpfung der sozialen Folgen der Covid-19-Pandemie dient, wurden die Auszahlungszeiten als vorübergehende Sonderregelung einmalig um drei Monate verlängert. Diese Verlängerung galt befristet für alle, deren Anspruch zwischen dem 1. Mai 2020 und dem 31. Dezember 2020 endete (Bundesgesetzblatt 2020).

**Table 1:** 

Versicherungspflicht in den letzten fünf Jahren vor Eintritt der Arbeitslosigkeit in Monaten	Vollendetes Lebensjahr	Maximale Bezugsdauer in Monaten	Covid-19-Pandemie-bedingt befristet bis 31.12.2020 verlängert in Monaten
12	–	6	+3
16	–	8	+3
20	–	10	+3
24	–	12	+3
30	50	15	+3
36	55	18	+3
48	58	24	+3

*Quelle*: eigene Darstellung nach BMAS(Bundesministerium für Arbeit und Soziales) (2020) und Bundesgesetzblatt (2020)

Wer das 50. Lebensjahr noch nicht vollendet hat und in den letzten fünf Jahren mindestens 24 Monate sozialversicherungspflichtig beschäftigt war, hat nach den Bestimmungen ohne die pandemiebedingten Erweiterungen Anspruch auf maximal 12 Monate Arbeitslosengeld. Zwischen dem 50. und dem 55. Lebensjahr steigt die Höchstbezugsdauer auf 15 Monate, ab Vollendung des 55. Lebensjahres bis zum 58. Lebensjahr auf 18 Monate und ab dem 58. Lebensjahr sind maximal 24 Monate Arbeitslosengeldbezug möglich. Die Lohnersatzrate beträgt 60 % des letzten Nettolohns, bei Existenz von Kindern nach dem Einkommensteuergesetz 67 %.

Während die Ersatzquote in den letzten Jahrzehnten der Transformation des Wohlfahrtsstaates relativ stabil geblieben ist, wurden wiederholt kontroverse Diskussionen über die maximale Dauer des Leistungsbezugs geführt. Seit Mitte der 1980er-Jahre wurden die Anspruchsrechte älterer Antragstellender schrittweise erweitert. Ältere Antragstellende hatten Anspruch auf Leistungen für höchstens 18–32 Monate, wobei die Höchstdauer von 32 Monaten für Personen ab 54 Jahren galt (seit 1997: ab 57 Jahren). Im Jahr 2006 wurde die maximale Dauer des Leistungsanspruchs auf 18 Monate verkürzt (Dlugosz et al. 2014). Nach einer intensiven Diskussion wurden die maximalen Bezugsdauern im Jahr 2008 für Ältere wieder auf die oben erläuterten aktuellen Bezugsdauern angehoben (Bothfeld und Rosenthal 2014, S. 203).

Immer wieder wurden, auch schon vor der Pandemie, Verlängerungen der maximalen Bezugsdauer vorgeschlagen. So treten etwa DIE LINKE und die SPD für eine Verlängerung der maximalen Leistungsdauer für Antragstellende mit langen Beitragsnachweisen ein, um ihre „Lebensleistungen“ zu respektieren (DIE LINKE 2019; SPD 2019). Die SPD schlägt vor, die maximale Leistungsdauer um drei, sechs oder neun Monate zu erhöhen, wenn Personen mindestens 20, 25 oder 30 Jahre lang Beiträge zur Arbeitslosenversicherung geleistet haben (SPD 2019). Der DGB schlägt vor, Arbeitslose sollten „für je zwei Beschäftigungsjahre einen zusätzlichen Monat Arbeitslosengeld erhalten (2:1-Regel). Wer beispielsweise insgesamt 20 Jahre sozialversicherungspflichtig gearbeitet hat, bekäme bis zu zehn Monate länger Arbeitslosengeld“ (DGB 2019, S. 3). Im DGB-Entwurf sollen bestimmte Zeiten der Kinderbetreuung und der Pflege von Angehörigen den Beschäftigungszeiten gleichgestellt werden. Mit der vorgeschlagenen Verlängerung sollen explizit „Arbeits- und Beitragsleistungen von Arbeitnehmerinnen und Arbeitnehmern stärker gewürdigt und anerkannt“ (SPD 2019) werden. Ob und, wenn ja, unter welchen Bedingungen die Bevölkerung eine solche Anerkennung von Lebensleistungen befürwortet, ist eine ebenso offene Frage wie mögliche Veränderungen von Einschätzungen in Krisenzeiten.

## Forschungsstand und Hypothesen

Wohlfahrtsstaatliche Institutionen greifen in individuelle Lebensverhältnisse ein und strukturieren soziale Beziehungen. Bürgerinnen und Bürger sind unmittelbar davon betroffen, wie Maßnahmen und Regelungen ausgestaltet sind: Als Empfängerinnen und Empfänger von Transferleistungen und als Adressaten sozialer Dienstleistungen ebenso wie als Beitrags- und Steuerzahlende, die zur Finanzierung des Sozialstaates beitragen. Für die Legitimität des Wohlfahrtsstaates ist es deshalb von Bedeutung, dass Bürgerinnen und Bürger auf politischer Ebene entschiedene Maßnahmen und Regelungen akzeptieren. Diese Akzeptanz von Wohlfahrtsprogrammen und -institutionen basiert auf Vorstellungen der Bevölkerung über ein als gerecht empfundenes Verhältnis zwischen Anstrengungen und Belohnungen und einem bestimmten Lebensstandard, den die Gesellschaft den Menschen wiederum als Gegenleistung für ihren Beitrag zur Gesellschaft ermöglichen soll (Bowles und Gintis 2000; Kaufmann 1997a; Mau 2004; Roosma et al. 2013). Um einen wohlfahrtsstaatlichen „Konsens“ oder die gesellschaftsintegrierende Funktion des Sozialstaates (Kaufmann 1997b) aufrechtzuerhalten, sollten also sozialpolitische Normen und institutionell festgelegte Allokations- und Verteilungsmechanismen als fair empfundene Verteilungsprinzipien widerspiegeln.

Diese Verteilungsprinzipien können sich dabei mehr oder weniger stark am Beitragsprinzip (Adams 1965) orientieren, d. h. an den Leistungen involvierter Personen (Greenberg 1990; Young 1993), ggf. verbunden mit dem Verantwortungsprinzip beeinflussbarer und nichtbeinflussbarer Ergebnisse (Konow 1996, 2001). Sie können aber auch mehr oder minder stark am Bedarf von einzelnen Betroffenen (Kluegel et al. 1999, S. 255; Gilliland 1993) ausgerichtet sein. Van Oorschot (2000) und van Oorschot und Roosma (2017) sprechen von Hilfewürdigkeit („deservingness“), die Anspruchsgruppen zugemessen wird und die von Bedarfen, geleisteten oder zukünftigen gesellschaftlichen Beiträgen oder von einer Einstellung, die Dankbarkeit signalisiert, beeinflusst sein kann (allgemein zu Gerechtigkeitsprinzipien auch Liebig 1997; Stephan et al. 2006, S. 21 ff.). Möglich sind aber auch generalisierte Abwertungen gegenüber Erwerbslosen (Freier 2016; Glatzer 2009; van Oorschot und Roosma 2015; Wogawa 2005; Zick 2010), die Auffassungen zu staatlichen Unterstützungen beeinflussen. Wogawa (2005) etwa sieht eine „Unbestimmtheitslücke“, die ein Misstrauen und Vorbehalte gegenüber eher großzügigen Regelungen dadurch erzeugen kann, dass individuelle Beweggründe einer Inanspruchnahme nicht vollständig zu erkennen und zu kontrollieren sind.

Vobruba (1996) hat darauf hingewiesen, dass in Gerechtigkeitsabwägungen zukünftige Wirkungen von Verteilungszuständen mitberücksichtigt werden. Diesem Tatbestand trägt „produktivistische Gerechtigkeit“ Rechnung (Vobruba 1996, S. 969; vgl. auch Leisering 1999, S. 11). Mit Blick auf die Forschung und Theorien zur Sozialpolitik werden damit direkt gesellschaftliche Effizienzwirkungen und ein „wirtschaftlicher Wert der Sozialpolitik“ (Vobruba 1991, S. 49 ff.; sowie Schmid 2009) angesprochen. Dies etwa dann, wenn Maßnahmen und Instrumente auf eine schnelle (Re‑)Integration in produktive Erwerbsarbeit ausgerichtet sind. Indirekt kann sich eine produktive Gerechtigkeit aber auch in einer Anerkennung von Arbeitsleistungen (in Erwerbsarbeit und Nichterwerbsarbeit) ausdrücken. Vorher erbrachte Leistung kann als Signal einer auch prospektiv zu erwartenden individuellen Leistungsfähigkeit und -bereitschaft interpretiert werden. Vor allem aber wird gegenüber Gesellschaftsmitgliedern ausgedrückt, dass sich jene, die Arbeitsleistungen erbringen, auf Leistungen des Sozialstaates verlassen können.

Gerechtigkeitsprinzipien, die sich eher an Beiträgen oder eher an Bedürfnissen orientieren, können ihrerseits unter Einbezug ihrer als (un-)produktiv eingeschätzten Wirkung beurteilt werden. Und so wird in Wissenschaft und Öffentlichkeit diskutiert, dass längere Bezugsdauern erstens Anreize für einen längeren Verbleib in Arbeitslosigkeit bieten (Kaufmann 2013; Mortensen 1986; Wogawa 2005). Zweitens kann diese längere Arbeitslosigkeit auch zu einem Abbau von Humankapital führen (so u. a. Addison und Portugal 2008; Caliendo et al. 2013; Katz und Meyer 1990; Riphahn und Schrader 2020; Schmieder et al. 2012, 2016; van Ours und Vodopivec 2008). Farber und Valletta (2015) zeigen, dass eben dieser Effekt auch in Krisen zu beobachten ist. Längere Lohnersatzleistungen bewirken, dass Arbeitslose sich mit der Suche nach einem gut passenden Arbeitsplatz länger Zeit lassen können (McCall 1970; Mortensen 1986). Diese Erkenntnis diente u. a. als Begründung dafür, dass im Zuge der sogenannten Hartz-Reformen eine Verkürzung des Arbeitslosengeldbezugs umgesetzt wurde. Dabei wurden solche Maßnahmen der Aktivierung Arbeitsloser auch öffentlich viel diskutiert (Kaufmann 2013). Jedoch können sehr kurze Bezugsdauern und ein schnell wirkender Zwang zur Arbeitsaufnahme die Qualität der Passung zwischen Beschäftigten und Stelle mindern (Belzil 1992, 2001; Caliendo et al. 2013; Ehrenberg und Oaxaca 1976; Voßemer und Schuck 2016; anders Fitzenberger und Wilke 2010 für ältere Beschäftigte).

Vor diesem Hintergrund leiten wir im Folgenden Hypothesen zur Akzeptanz der gesetzlichen Ausgestaltung von Bezugsdauern des Arbeitslosengeldes ab.

Grundsätzliche Gerechtigkeitsprinzipien variieren innerhalb stabiler Gesellschaften wenig (Gouldner 1960; Wegener 1992). Zudem ist die Ausgestaltung der Arbeitslosenversicherung u. a. auf konjunkturelle Krisen ausgerichtet. Dennoch könnte sich die Akzeptanz gegenüber spezifischen sozial- und arbeitsmarktpolitischen Normen, etwa aufgrund von sozioökonomischen Entwicklungen, verändern, und dies möglicherweise auch situationsbedingt kurzfristig. Wenn wir die Akzeptanz der gesetzlichen Ausgestaltung von Bezugsdauern bei Arbeitslosen betrachten, dann kann diese unter anderem davon beeinflusst sein, wie sich die Gesamtsituation auf dem Arbeitsmarkt darstellt und als wie schwer es von den Befragten eingeschätzt wird, dass arbeitslos gewordene Personen eine neue Arbeitsstelle finden.

In der ersten Hochphase der Covid-19-Pandemie bis zum Juni 2020 haben Arbeitgeber aus wirtschaftlichen Gründen vor allem das Instrument der Kurzarbeit genutzt. Darüber hinaus haben sie aber auch weniger neue Stellen ausgeschrieben, weniger Neueinstellungen vorgenommen und häufiger Beschäftigten gekündigt. Kündigungen waren zunächst auf wenige Branchen wie das Gastgewerbe konzentriert, die besonders hart von den politischen Maßnahmen zur Eindämmung der Pandemie betroffen waren (Bossler et al. 2020; Kubis 2020; Sauer und Wohlrabe 2020; Weber und Gehrke 2020). Diese wirtschaftliche Reaktion hatte verschiedene Ursachen: Mangelnde Zulieferungen und Verkäufe, Kontaktbeschränkungen zu Beschäftigten sowie Kundinnen und Kunden oder die Unsicherheit über die zukünftige Situation. In dieser Hochphase der pandemiebedingten Einschränkungen ist neueingetretene Arbeitslosigkeit oft nicht selbstverschuldet. Das Verantwortlichkeitsprinzips (Konow 1996, 2001) legt nahe, dass die Bevölkerung in der Krise tendenziell längere Bezugsdauern von Arbeitslosengeld befürwortet. Roosma et al. (2016) argumentieren in ähnlicher Weise, dass Personen in wirtschaftlich schwierigen Zeiten weniger Kontrolle über ihre Situation am Arbeitsmarkt haben und/oder ein größerer Bedarf nach Unterstützung plausibel ist (vgl. zu den Kriterien „Control“ und „Need“ auch van Oorschot 2000, S. 36).

### H 1

In Krisenzeiten werden Arbeitslosen längere Höchstbezugsdauern zugesprochen als in Nichtkrisenzeiten.

Aktuell steigt die maximale Arbeitslosengeldbezugsdauer von zwölf Monaten ab dem vollendeten 50. Lebensjahr in Schritten bis zu einer Höchstbezugsdauer von maximal 24 Monaten ab dem Alter von 58 Jahren. Dies spiegelt wider, dass Ältere es – ohne eigenes Verschulden – schwerer haben, bei Arbeitslosigkeit einen neuen Arbeitsplatz zu finden. Sie haben weniger Verantwortung für (Konow 1996, 2001) und Kontrolle über ihre Situation als jüngere Arbeitslose und entsprechend eine andere Bedürftigkeit.

### H 2a

Älteren Arbeitslosen werden längere Bezugsdauern des Arbeitslosengeldes zugesprochen als etwas jüngeren Arbeitslosen.

In wirtschaftlichen Krisenzeiten und in der Covid-19-Pandemie ist es für alle Altersgruppen schwer, eine neue Stelle zu finden. Wir vermuten, dass Befragte dieses in ihren Urteilen berücksichtigen.

### H 2b

Während der Pandemie werden älteren und etwas jüngeren Arbeitslosen gleiche Bezugsdauern des Arbeitslosengeldes zugesprochen.

Arbeitslosen, denen eine individuelle Mitverantwortung für eine Entlassung zugemessen wird, werden vermutlich eher kürzere Bezugsdauern zugestanden. Das kann dadurch beeinflusst sein, dass sich das Gerechtigkeitsempfinden auch an Eigenschaften und Verhaltensweisen der Akteure orientiert (van Oorschot 2000; Buss 2019). In diesem Fall haben Personen, denen eine Mitschuld an ihrer Arbeitslosigkeit attestiert wird, Kontrolle über die Situation.

### H 3a

Besteht eine Mitverantwortung an der Arbeitslosigkeit, dann werden Arbeitslosen geringere Bezugsdauern zugesprochen.

In einer Krise bestehen geringe Wiederbeschäftigungschancen. Eigenkündigungen und andere selbstverschuldete Arbeitslosigkeit sind riskant und können von Befragten als fahrlässig oder verantwortungslos bewertet werden. Vor diesem Hintergrund vermuten wir:

### H 3b

Ist Arbeitslosigkeit selbst mitverschuldet, so werden Arbeitslosen während der Covid-19-Pandemie noch geringere Bezugsdauern als vor der Krise zugesprochen.

Das Beitragsprinzip nach Adams (1965) umfasst einzelne individuelle Leistungen auf hierauf beziehbare Ergebnisse. Die Arbeitslosenversicherung folgt einem eng an der Lohnerwerbsarbeit orientiertem Beitragsprinzip. Allerdings bestimmt sich die maximale Anspruchsdauer aktuell u. a. anhand der versicherungspflichtigen Zeiten in den letzten fünf Jahren. Wenn Fairnesswahrnehmungen sich am Beitragsprinzip ausrichten, dann ist es plausibel anzunehmen, dass diejenigen, die in der Vergangenheit längere Zeit zum System beigetragen haben, in Zukunft längere Zahlungen erhalten sollten. In diesem Fall würde den entsprechenden Personen auch ein höheres Ausmaß an Reziprozität zugestanden (van Oorschot 2000, S. 36).

### H 4a

Dauerhaft Beschäftigten mit regelmäßigen Beiträgen in die Sozialversicherung werden eher längere maximale Bezugsdauern zugemessen als unregelmäßig Beschäftigten.

Eine grundlegende Orientierung an Pflichten (hier längere Einzahlungen in die Arbeitslosenversicherung) zur Erlangung von Rechten (hier umfänglichere Ansprüche an Leistungen des sozialen Sicherungssystems) ist u. a. in Deutschland in normativen Leitbildern und Einstellungen stark verankert (Sachweh et al. 2006, 2009; Vobruba 1989). Deshalb vermuten wir:

### H 4b

Ein Reziprozitäts- oder Betragseffekt ist in gleicher Stärke auch in der Pandemiekrise zu beobachten.

Beiträge oder Leistungen können sich aber auch allgemeiner auf die Aufrechterhaltung gesellschaftlicher Funktionen richten, die dann wiederum einer Gesellschaft und ihren Mitgliedern dienlich sind und von der Gesellschaft auch honoriert werden. Wohlfahrten basieren auf solchen Reziprozitäten (Bowles und Gintis 2000; Kaufmann 1997a; Mau 2004). Entsprechend wäre es im Sinne eines an reziproken Beiträgen orientierten Prinzips möglich, dass die Bevölkerung bei Leistungen aus der Arbeitslosenversicherung auch andere Arbeitsformen, etwa gesellschaftlich relevante Sorgearbeiten, und nicht allein die Aufnahme einer Erwerbstätigkeit in die Beurteilung miteinbezieht. Die Wohlfahrtsstaatdebatte greift den Aspekt auf, dass Sozialversicherungen und insbesondere die Arbeitslosen- und Rentenversicherung auf den „normalen“ Lebensverlauf einer kontinuierlichen Vollzeitbeschäftigung ausgerichtet sind. Menschen mit einer diskontinuierlichen Erwerbsbiografie oder Teilzeitarbeit sind nicht in gleichem Maße abgesichert. Auch Herausforderungen, die sich aus der Vereinbarkeit von Beschäftigung, Familienleben sowie lebenslangem Lernen ergeben, fängt das soziale Netz sozial- und arbeitsmarktpolitisch nur begrenzt auf (Seifert und Struck 2009; Vobruba 1989).

### H 5a

Personen, die neben der Lohnerwerbsarbeit „gesellschaftlich nützliche“ Tätigkeiten verrichten, werden längere maximale Bezugsdauern zugemessen als Personen, bei denen keine Hinweise hierauf vorliegen.

### H 5b

Ein solcher Effekt ist in der Pandemiekrise in gleicher Stärke zu beobachten.

Sozialversicherungen sind im Allgemeinen stark an Beiträge aus Erwerbsarbeit gekoppelt. Ein Äquivalenzprinzip gilt besonders auch für die an die individuelle Einkommenshöhe gekoppelte Arbeitslosenversicherung. Doch in geringerem Maße institutionalisiert auch die Arbeitslosenversicherung bedarfsgerechte finanzielle Leistungen im Hinblick auf die Leistungshöhe: Antragstellende mit unterhaltsberechtigten Kindern erhalten bei Arbeitslosigkeit einen höheren Anteil ihres bisherigen Nettoeinkommens (67 vs. 60 %). Für die Dauer des Arbeitslosengeldbezugs sind Bedarfsaspekte jedoch unerheblich. Insgesamt vermuten wir, dass Tatbestände der Bedürftigkeit nicht beeinflussen, welche Bezugsdauer des Arbeitslosengeldes als angemessen angesehen wird. Bedürftigkeit wird in der Analyse dabei über den Beitrag des Partners oder der Partnerin zum Haushaltseinkommen operationalisiert.

### H 6a

Finanziell bedürftigeren Arbeitslosen wird keine längere maximale Bezugsdauer gewährt als weniger bedürftigen Arbeitslosen.

Die Pandemie und der wirtschaftliche Lockdown im April/Mai 2020 hatten erhebliche Unsicherheit über die wirtschaftliche Situation vieler Haushalte zur Folge. Wir vermuten, dass Befragte ihre Urteile zu Leistungen in Krisenzeiten generöser auf finanzielle Bedarfe und die Höchstdauern ihrer Gewährung ausrichten als vor der Pandemie.

### H 6b

Finanziell bedürftigeren Arbeitslosen wird in der Pandemiekrise eine längere maximale Bezugsdauer gewährt als in Nichtkrisenzeiten.

Schließlich sollte unabhängig von der Befragungszeit vor oder während der Pandemiekrise gelten, dass in Gesellschaften, in denen ein grundsätzlicher Konsens über sozialpolitische Verteilungsprinzipien besteht, ein Hinweis auf die spezifischen gesetzlichen Normen von vielen tatsächlich als „Anker“ akzeptiert wird und viele Befragte ihre Angaben zur Höchstbezugsdauer von Arbeitslosengeld noch näher an den gesetzlichen Normen ausrichten, wenn sie einen derartigen Anker erhalten.

### H 7

Wird in der Befragung auf die Gesetzeslage hingewiesen, dann orientieren sich Personen bei ihren Angaben eher an diesen Normen.

## Datengrundlage und methodisches Vorgehen

Die Auswertungen basieren auf zwei Onlinebefragungen. Die befragten Personen wurden aus einer 2 %-Stichprobe der „Integrierten Erwerbsbiografien“ (IEB) (IEB V13.01.00-181010) ausgewählt (Osiander et al. 2020). Die IEB umfassen Zeiten registrierter Arbeitssuche und Arbeitslosigkeit, des Bezugs von Arbeitslosengeld oder Arbeitslosengeld II, der Maßnahmenteilnahme und der Beschäftigung (siehe Antoni et al. 2019 für eine Beschreibung der schwach anonymisierten Version der IEB).

Den hier präsentierten Analysen liegt eine Stichprobe vergleichsweiser arbeitsmarktnaher Personen zugrunde, die im Jahr 2017 mindestens eine Meldung in den IEB hatten und die in den Jahren 2013–2017 ausschließlich sozialversicherungspflichtig oder geringfügig beschäftigt waren (aber keine Zeiten der Arbeitsuche, des Leistungsbezugs oder der Teilnahme an Maßnahmen aufwiesen). Die Stichprobe wurde weiterhin auf deutsche Staatsbürger eingegrenzt, die zum Zeitpunkt der Befragung mindestens 18 Jahre alt waren, nicht im Ausland lebten und für die relevante soziodemografische und betriebsspezifische Informationen vorlagen.

Potenzielle Befragte dieser Gruppe – insgesamt 19.934 Personen – erhielten ein postalisches Anschreiben mit einem individualisierten Link zur Befragung und ein Passwort sowie einen QR-Code mit Link zur Befragungsseite. Der faktorielle Survey, der die Frage zur Angemessenheit der maximalen Bezugsdauer enthält, wurde zufällig der Hälfte der Angeschriebenen vorgelegt (die andere Hälfte erhielt inhaltlich anders gelagerte Themen).

In Anlehnung an die AAPOR-Richtlinie (2016) berechnen wir die Nettorücklaufquote konservativ. 1324 Personen oder 6,6 % aller postalisch angeschriebenen Personen beantworteten die erste Befragung vollständig (Osiander et al. 2020, Tab. 1, Spalte 3). Dies liegt im Rahmen dessen, was zu erwarten war. Die Teilnehmerstichproben sind nicht repräsentativ für die deutsche Erwerbsbevölkerung. Allerdings ermöglicht es die Ziehung aus den IEB, Selektivitäten umfassend nachzuvollziehen. Eine Selektivitätsanalyse für alle postalisch kontaktierten Personen zeigt (Osiander et al. 2020, Tab. 4): Knapp 50 % der Befragungspersonen der Bruttostichprobe sind weiblich, 15 % leben in Ostdeutschland; 74 % haben eine abgeschlossene berufliche Ausbildung und 21 % einen (Fach‑)Hochschulabschluss. Im Vergleich zur Bruttostichprobe sind Frauen, Personen aus Ostdeutschland und Personen ab einem Alter von 30 Jahren geringfügig unterrepräsentiert. Formal besser Qualifizierte, Personen mit längeren Beschäftigungsdauern oder Arbeitslosengeldbezug in der Vergangenheit nahmen etwas häufiger an der Befragung teil.

Aus dem Nettosample der ersten Befragung wurden diejenigen 634 Teilnehmenden der ersten Befragung, die die Vignetten zur Bezugsdauer des Arbeitslosengeldes erhalten hatten, im Mai 2020 – also in einer Hochphase pandemiebedingter Einschränkungen – erneut per Post angeschrieben und zu einer Wiederholungsbefragung eingeladen. Von den 634 angeschriebenen Personen beantworteten 227 Personen den zweiten Onlinefragebogen, 201 davon vollständig. Multivariate Selektivitätsanalysen^1^ zeigen hier, dass im Vergleich zur ersten Befragungswelle Personen mit sehr hohem Einkommen von 5000 € oder mehr überrepräsentiert sind. Personen, die jünger als 29 Jahre oder zwischen 30 und 39 Jahre alt sind, sind unterrepräsentiert (siehe Tabelle 5 im Anhang). Die Befragung wurde durchgeführt während der Bundestag die Verlängerung des Arbeitslosengeldes im Rahmen des von der Bunderegierung im April 2020 vorgelegten Sozialschutzpakets II verabschiedete. Rückwirkend trat diese Änderung dann zum 01.05.2020 in Kraft. Die weiteren Analysen führen wir mit einem Sample von 186 Personen durch, die der Verknüpfung ihrer Daten über die Befragungswellen hinweg zugestimmt haben, die zu beiden Befragungszeitpunkten Angaben zu den Vignetten machten und für die weitere soziodemografische und Einstellungsvariablen vorliegen. Im Wesentlichen ist diese Reduktion der Fallzahlen auf Befragte zurückzuführen, die die Vignetten nicht zu beiden Zeitpunkten beantwortet haben.

Methodisch baut die Untersuchung auf einem faktoriellen Survey auf (einführend z. B. Auspurg und Hinz 2015; Beck und Opp 2001). Faktorielle Surveys bestehen aus mehreren sogenannten Vignetten. Hierbei handelt es sich um hypothetische Szenarien, die Befragten zur Beurteilung vorgelegt werden – oft anhand von Skalen, teilweise mit der Bitte um die Angabe von Zahlenwerten. Zentrales Merkmal von Vignetten ist, dass wesentliche Charakteristika des jeweiligen Szenarios ähnlich wie in einem Experiment zufällig variiert werden. Von diesen Charakteristika wird auf Basis theoretischer Überlegungen angenommen, dass sie das Urteil maßgeblich beeinflussen. Die Befragten beurteilen in der Regel mehrere Szenarien. Vignetten haben sich in unterschiedlichen Forschungskontexten als geeignete Untersuchungsmethode erwiesen (Auspurg und Hinz 2015; Finch 1987; Wallander 2009). Die zufällige Variation der Ausprägungen erlaubt es, die Effekte unterschiedlicher Merkmale auf das Antwortverhalten isoliert voneinander zu analysieren, selbst wenn diese Merkmale in der Realität hochkorreliert auftreten.

Unser faktorieller Survey zur Bezugsdauer des Arbeitslosengeldes beschreibt eine Person, die seit Kurzem arbeitslos ist und die die in Tab. 2 dargestellten Merkmale aufweist.

**Table 2:** 

Dimension	Ausprägungen	Anzahl Ausprägungen
Geschlecht	Männlich	2
	Weiblich	
Alter in Jahren	48	4
	52	
	56	
	60	
Grund der Arbeitslosigkeit	Konkurs des Arbeitgebers	2
	Vertrag aufgrund häufiger Fehlzeiten nicht verlängert	
Beschäftigung in der Vergangenheit	Dauerhaft	2
	Unregelmäßig	
Beiträge zum Allgemeinwesen	Keine Kinder	3
	Zwei erwachsene Kinder	
	Pflegebedürftiger Vater	
Beitrag des Partners zum Haushaltseinkommen	Kein Einkommen des Partners	3
	Kann Haushaltsbedarf teilweise decken	
	Kann Haushaltsbedarf vollständig decken	

*Quelle*: eigene Darstellung

Die beschriebene Person ist entweder männlich oder weiblich. Die vier gewählten Altersangaben von 48, 52, 56 und 60 Jahren wurden aufgrund der aktuell gültigen Sprungstellen der maximalen Bezugsdauer (bei 50, 55 und 58 Jahren) gewählt. Als Kündigungsgrund ist entweder ein durch den Einzelnen nicht kontrollierbares Ereignis – der Konkurs des Arbeitgebers – angegeben oder das Auslaufen des Arbeitsvertrags, das durch das Fehlverhalten der Person selbst mitbeeinflusst wurde („häufig zu spät gekommen“).^2^ Die Person hat in der Vergangenheit durch regelmäßige oder unregelmäßige Beschäftigung entweder dauerhaft oder nur phasenweise Beiträge zur Arbeitslosenversicherung gezahlt. Sie hat zudem in verschiedener Form gesellschaftlich nützliche Beiträge erbracht (oder eben nicht): Die Person ist entweder kinderlos, hat zwei erwachsene Kinder oder kümmert sich um ihren pflegebedürftigen Vater. Die beschriebene Person hat zudem eine unterschiedlich ausgeprägte individuelle Bedürftigkeit: Der Lebenspartner oder die Lebenspartnerin hat entweder kein eigenes Einkommen oder kann den Haushaltsbedarf teilweise oder auch vollständig decken. Das Vignettenuniversum – alle möglichen Kombinationen der Ausprägungen – besteht aus 2 * 4 * 2 * 2 * 3 * 3 = 288 Kombinationsmöglichkeiten. Wir verwenden das gesamte Universum, ohne Fälle auszuschließen (Full Factorial Design). Unlogische oder sehr unwahrscheinliche Kombinationen kommen nicht vor. Wir unterteilen das Vignettenuniversum in 72 Vignettendecks. Die Decks werden zufällig aus den Vignetten generiert. Die Reihenfolge der Vignetten innerhalb des Decks ist ebenfalls zufällig. Eine ausformulierte Beispielvignette findet sich nachstehend. Die kursiven Teile waren auch für die Befragten optisch hervorgehoben.

Eine *48-jährige (48-jähriger, 52-jährige/r, 56-jährige/r, 60-jährige/r) Frau (Mann)* ist seit Kurzem arbeitslos. Ihr (Sein) Arbeitsvertrag wurde nicht verlängert, *weil sie (er) häufig zu spät kam*. (Das Unternehmen, in dem sie/er gearbeitet hat, musste *wegen finanzieller Schwierigkeiten *aufgeben.) Sie (Er) war seit ihrem (seinem) 22. Lebensjahr *dauerhaft (unregelmäßig) beschäftigt* und hat (*phasenweise*) Beiträge in die Arbeitslosenversicherung eingezahlt. Sie (Er) kümmert sich um ihren *pflegebedürftigen Vater*. (Sie/Er hat* keine/zwei erwachsene Kinder*.) Ihr Mann (Seine Frau) kann durch sein (ihr) Einkommen den Haushaltsbedarf *teilweise (vollständig) decken*. (Ihr Mann/Seine Frau hat im Moment *kein Einkommen*.)

Die Befragten wurden gebeten, anzugeben, wie lange die beschriebene Person ihrer Ansicht nach *maximal* Arbeitslosengeld erhalten sollte. Dazu wurde ein Freitextfeld eingeblendet, in das die Zahl der Monate eingetragen werden konnte. Dabei wurde der Bereich auf die Werte zwischen 0 und 99 Monate eingeschränkt. In der erneuten Befragung im Mai 2020 wurde die Abfrage der abhängigen Variable modifiziert und erweitert. Die Befragten sollten angeben, wie lange die beschriebene Person „in normalen Zeiten (wie vor der Coronakrise)“ und „in der aktuellen Coronakrise“ maximal Arbeitslosengeld beziehen sollte. Für den Vergleich verwenden wir im Folgenden die Angaben aus dem November 2019 und die Angaben zu „in der aktuellen Coronakrise“ vom Mai 2020.^3^

Die Befragten wurden in beiden Befragungen gebeten, jeweils vier Vignetten zum Arbeitslosengeld zu beantworten, die zu beiden Zeitpunkten identisch waren. In der zweiten Befragungswelle sind die einzelnen Decks etwas ungleicher verteilt als in der ersten. Jedoch gibt es in beiden Befragungswellen keine signifikanten Korrelationen (α = 0,05) der Vignettendimensionen untereinander. Das spricht dafür, dass die zufällige Zuweisung von Vignetten zu Personen in beiden Wellen erfolgreich funktioniert hat. Bei etwa der Hälfte der Befragten wurde vor den Vignetten zufällig der in Tab. 3 dargestellte Hinweistext eingeblendet, der über die tatsächlichen gesetzlichen Regelungen zum Zeitpunkt der Befragung informiert.

**Table 3:** 

„Sozialversicherungspflichtig Beschäftigte zahlen in Deutschland Beiträge zur Arbeitslosenversicherung. Wer Arbeitslosengeld beantragen möchte, muss sich bei der Arbeitsagentur arbeitslos melden. Wie lange maximal Arbeitslosengeld bezogen werden kann, ist abhängig vom Alter: Personen …	
… bis zu einem Alter von 49 Jahren	… erhalten bis zu 12 Monate Arbeitslosengeld
… im Alter zwischen 50 und 54 Jahren	… erhalten bis zu 15 Monate Arbeitslosengeld
… im Alter zwischen 55 und 57 Jahren	… erhalten bis zu 18 Monate Arbeitslosengeld
… ab einem Alter von 58 Jahren	… erhalten bis zu 24 Monate Arbeitslosengeld.“

*Quelle*: eigene Darstellung

Wir gehen davon aus, dass nicht jede oder jeder Befragte im Detail über die gesetzlichen Regelungen informiert war und der Hinweis deswegen für viele Befragten eine zusätzliche Information bereitstellt. Durch dieses Vorgehen lässt sich prüfen, inwieweit Ankereffekte die Einschätzungen der Befragten in ihrem Niveau und ihrer Streuung beeinflussen. Wir gehen entsprechend Hypothese *H 7* davon aus, dass Personen, die den Hinweis eingeblendet bekamen, aus Gründen des Einvernehmens mit wohlfahrtstaatlichen Prinzipien ihre Einschätzung eher an der aktuellen gesetzlichen Lage ausrichten. In der Wiederholungsbefragung im Mai 2020 wurde der Hinweis um folgenden Satz ergänzt: „Aufgrund der Coronakrise wurde die maximale Bezugsdauer kürzlich unter bestimmten Voraussetzungen verlängert.“

Alle Befragten wurden in der ersten Befragung im Herbst 2019 um einige zusätzliche soziodemografischen Informationen gebeten (eigene Kinder, Anzahl Personen im Haushalt, klassiertes Nettohaushaltseinkommen). Anhand der Frage, welcher Partei sie am nächsten stehen, wurde zudem die politische Orientierung erfasst. Der Fragebogen enthält außerdem zwei Items, die die eigene Einstellung zur Schuld an Arbeitslosigkeit abfragen. Letztere sind in abgewandelter Form von Boockmann et al. (2014) übernommen. Erwerbstätige Personen wurden gefragt, für wie wahrscheinlich sie es halten, in den nächsten 12 Monaten (zeitweise) arbeitslos zu sein.

Im Folgenden berichten wir die Ergebnisse der beiden Befragungen im Vergleich und eine multivariate Analyse der Einflussfaktoren auf die von den Befragten als angemessen empfundene Bezugsdauer von Arbeitslosengeld.

## Ergebnisse

Zum Befragungszeitpunkt im November 2019 geben die Befragten im Durchschnitt 22,6 Monate als angemessene Bezugsdauer an. Dieser Wert liegt für die spezifischen beschriebenen Situationen über den gesetzlich festgelegten maximalen Bezugsdauern. Im Mai 2020 halten sie in der aktuellen Coronakrise 22,0 Monate für angemessen, also sogar etwas weniger als zuvor. Ein t‑Test auf Mittelwertunterschiede zwischen den Gruppen lehnt die Nullhypothese gleicher Mittelwerte nicht ab (*p* = 0,14). Entgegen unserer Vermutung in *H 1* sehen die Befragten zu dem untersuchten Krisenzeitpunkt im Mai 2020 längere Bezugsdauern nicht als gerechtfertigt an.

**Figure 1:**
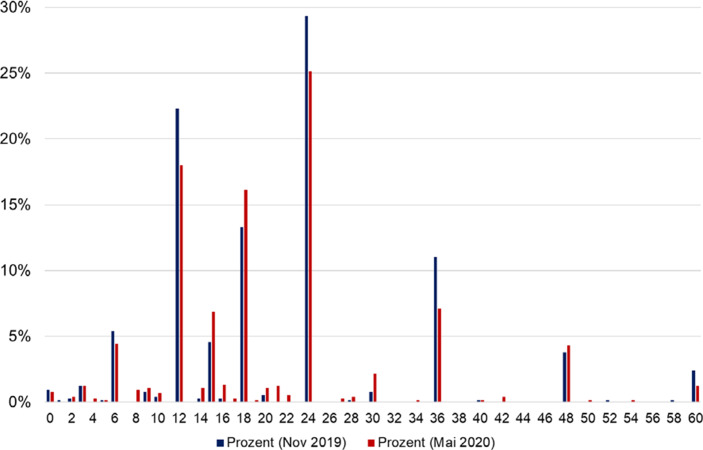

Abbildung 1 zeigt die Verteilung der als angemessen erachteten maximalen Bezugsdauern zu beiden Messzeitpunkten. Beide Male wählten die Befragten am häufigsten 24 Monate als angemessene Länge – im November 2019 in knapp 30 % und im Mai 2020 in etwa 25 % der Fälle. Am zweithäufigsten wird eine Bezugsdauer von 12 Monaten genannt (Nov. 2019: 22 %, Mai 2020: 18 %), gefolgt von 18 Monaten (Nov. 2019: 13 %, Mai 2020: 16 %).

**Figure 2:**
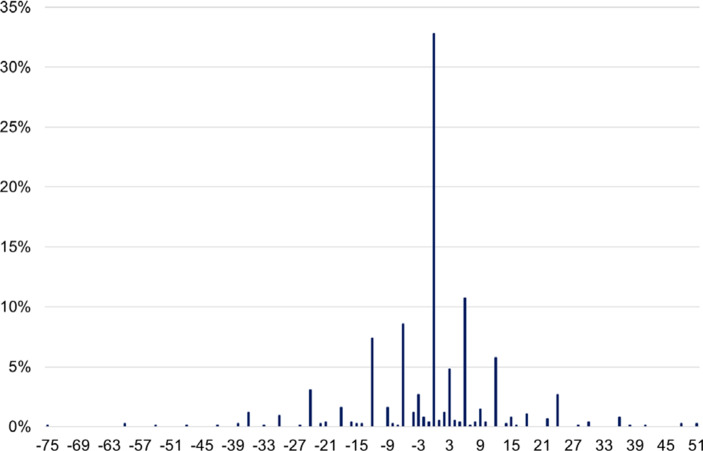

Abbildung 2 stellt die Verteilung der individuellen Differenzen bei den einzelnen Szenarien zwischen beiden Befragungszeitpunkten dar. In etwa einem Drittel aller Fälle ist die Differenz zwischen den beiden Befragungszeitpunkten 0, d. h. die Befragten ändern ihr Urteil zwischen den Befragungszeitpunkten nicht. Relativ häufig werden Änderungen um sechs oder zwölf Monate vorgeschlagen, sowohl in Form von Kürzungen der Bezugsdauern als auch in Form von Verlängerungen.

Tabelle 4 zeigt die Ergebnisse einer multivariaten Analyse der Einflussfaktoren auf die als angemessen erachtete maximale Bezugsdauer und inwieweit sich diese in der Coronakrise im Mai 2020 im Vergleich zu vor der Krise verändert haben. Es ist davon auszugehen, dass die Einschätzungen unterschiedlicher Szenarien durch eine Person nicht unabhängig voneinander sind. Dies muss durch das Schätzmodell berücksichtigt werden. Wir präsentieren im Folgenden die Ergebnisse von Random-Effects-Modellen.^4^

**Table 4:** 

	RE-Modell 1: Max. Bezugsdauern		RE-Modell 2: Differenz der max. Bezugsdauern^#^	
Vignettendimension	Koeffizient	Std.- Fehler	Koeffizient	Std.- Fehler
Dummy für Befragung während Corona (Ref.: vor Corona)	−0,64	0,83	–	–
Mann (Ref.: Frau)	−0,05	0,43	−0,06	0,672
48 Jahre (Ref.)	–	–	–	–
52 Jahre	1,86**	0,60	0,04	0,93
56 Jahre	2,87***	0,70	−0,70	0,87
60 Jahre	5,79***	0,74	−0,09	0,95
Konkurs des Arbeitgebers (Ref.: Vertrag wegen häufiger Verspätung nicht verlängert)	3,14***	0,43	−0,43	0,65
In der Vergangenheit durchgängig beschäftigt (Ref.: unregelmäßig beschäftigt)	4,31***	0,58	−2,52**	0,68
Keine Kinder (Ref.)	–	–	–	–
Zwei erwachsene Kinder	1,07	0,52	−0,61	0,86
Pflegebedürftiger Vater	2,66***	0,57	−0,76	0,82
Partner/in hat kein Einkommen (Ref.)	–	–	–	–
Partner/in kann Bedarf des Haushalts teilweise decken	−1,30*	0,60	0,34	0,81
Partner/in kann Bedarf des Haushalts vollständig decken	−4,01***	0,67	1,27	0,80
Hinweis zur aktuellen Rechtslage (Ref.: kein Hinweis)	−0,78	1,68	–	–
Konstante	17,24	1,73	0,38	1,40
*Personen*	186		186	
*Beobachtungen*	1488		744	
*Pseudo-R2*	0,08		0,02	

*Quelle*: eigene Berechnungen

Legende: *** *p* < 0,001; ** *p* < 0,01; * *p* < 0,05; ^+^ *p* < 0,10

^#^ Angabe im Mai 2020 abzüglich Angabe im November 2019

Es wurde zusätzlich für die Vignettenposition kontrolliert. Da es an den Rändern der Verteilung der Residuen zu leichten Abweichungen von einer Normalverteilung kommt, sind in den Schätzungen robuste Standardfehler ausgewiesen

Im RE-Modell 1 sind die Determinanden der angegebenen maximalen Bezugsdauern ausgewiesen. Für die Befragung während der Covid-19-Pandemie wird eine Dummyvariable aufgenommen. Eine weitere Random-Effects-Schätzung (RE-Modell 2) regressiert die Differenz der angegebenen Bezugsdauern zwischen den beiden Befragungszeitpunkten auf die Vignettendimensionen.^5^ Inhaltlich analysieren wir damit, welchen beschriebenen Personen während der Pandemie systematisch längere oder kürzere Bezugsdauern zugestanden werden. Im Folgenden werden die Ergebnisse entsprechend der Hypothesen mit Blick auf die Schätzergebnisse jeweils beider Modelle vorgestellt.

Die Vignettendimensionen haben überwiegend zu erwartende Effekte. Beiden Geschlechtern werden ähnliche Bezugsdauern zugestanden. Mit zunehmendem Alter der beschriebenen Person würden die Befragten erwartungsgemäß längere Bezugsdauern gewähren (*H2a*). Im Vergleich zur 48-jährigen Referenzperson wird einer 52-jährigen Person im Durchschnitt eine um knapp zwei Monate längere Bezugsdauer zugestanden (*p* < 0,01), einer 56-jährigen Person eine um knapp drei Monate längere und einer 60-jährigen Person eine um knapp sechs Monate längere (*p* < 0,001).^6^ Der Effekt des hohen Erwerbsalters von 60 Jahren ist zudem der stärkste im gesamten Modell. Dennoch differenzieren die Befragten letztlich weniger stark als der Gesetzgeber, der 60-Jährigen aktuell bis zu zwölf Monate länger Arbeitslosengeld gewährt als 48-Jährigen. Allerdings werden mit Personen zwischen 48 und 60 Jahren solche abgebildet, die mitten oder in der zweiten Hälfte ihres Erwerbslebens stehen. Die Effekte des Alters würden evtl. noch deutlicher ausfallen, wenn jüngere hypothetische Personen miteinbezogen worden wären.

Die Effekte der zugemessenen längeren Bezugsdauern zugunsten Älterer erhalten sich auch in der Krise (RE-Modell 2). Hierbei ist, entgegen der Hypothese *H2b* keine stärker als in Nichtkrisenzeiten nach Alter differenzierte Abstufung der zugesprochenen Bezugsdauern zu beobachten.

Gegenüber Personen, die durch einen Unternehmenskonkurs arbeitslos geworden sind, sind die Befragten generöser als gegenüber Personen, deren Vertrag aufgrund wiederholter Unpünktlichkeit nicht verlängert wurde (+3 Monate, *p* < 0,001). Ein Effekt, der auch in der Covid-19-Pandemie in vergleichbarer Höhe besteht (RE-Modell 2). Das steht im Einklang mit Hypothese *H 3a*. Wir hatten aber in *H 3b* erwartet, dass Mitverantwortung an Arbeitslosigkeit in der Krise mit größerer Wahrscheinlichkeit „sanktioniert“ wird. Die zeigt sich nicht. Ähnliches gilt für Personen, die in der Vergangenheit durchgängig beschäftigt waren und deshalb permanent Beiträge ins System eingezahlt haben. Die Befragten gestehen diesem Personenkreis in Nichtkrisenzeiten einen etwa vier Monate längeren Leistungsbezug zu als Personen, die unregelmäßig beschäftigt waren (*p* < 0,001). Das stimmt mit den in Hypothese *H 4a* formulierten Erwartungen überein. Wir hatten zudem erwartet, dass ein solcher Äquivalenz- oder Beitragseffekt (Adams 1965; van Oorschot 2000, S. 36) auch in der Krise erhalten bleibt (*H 4b*). Für Personen, die durchgängig beschäftigt waren, verringert sich jedoch der Unterschied im Vergleich zu den in Vignetten als unregelmäßig beschäftigt ausgewiesenen Personen in der Pandemie um 2,5 Monate (*p* < 0,01). Entsprechend wird mit höherer Wahrscheinlichkeit angegeben, dass durchgängig Beschäftigten in der Pandemie etwas kürzere Leistungsbezüge zugemessen werden. Dies könnte darauf hinweisen, dass Teile der Bevölkerung in Krisenzeiten das Beitragsprinzip der Arbeitslosenversicherung als etwas weniger wichtig ansehen als in Nichtkrisenzeiten (Bowles und Gintis 2000; Mau 2004; Sachweh et al. 2009; Vobruba 1989).

Im Vergleich mit Personen, die keine Kinder haben, gewähren die Befragten Personen, die aktuell den eigenen Vater pflegen, einen leicht längeren Arbeitslosengeldbezug von etwa 2,5 Monaten (*p* < 0,001). Die in Hypothese *H 5a* und *H 5b* formulierten Erwartungen, dass gesellschaftlich nützliche Tätigkeiten vor und in der Krisenzeit honoriert werden, bestätigt sich damit.

Personen, deren Partnerin oder Partner durch ihr oder sein Einkommen den finanziellen Bedarf des Haushalts teilweise oder vollständig decken kann, werden von den Befragten hingegen restriktiver behandelt. Ist der Bedarf teilweise gedeckt, kürzen die Befragten den Leistungsbezug um gut einen Monat (*p* < 0,05) im Vergleich zur Situation, in der die Partnerin oder der Partner kein eigenes Einkommen hat. Kann die andere Person im Haushalt den finanziellen Bedarf vollständig decken, wird die Leistung um vier Monate gekürzt (*p* < 0,001). Dies widerspricht den Hypothesen *H 6a* und *H 6b*, in denen wir erwarten, dass finanziell bedürftigen Arbeitslosen in Nichtkrisenzeiten keine längeren Bezugsdauern gewährt werden, in Krisenzeiten jedoch höhere Bedarfe auch über Mittel der beitragsfinanzierten Arbeitslosenversicherung positiv berücksichtigt werden. Letzteres ist der Fall, aber es besteht keine Differenz zwischen Krise und Nichtkrise.

Ein Hinweis auf die aktuelle Rechtslage als Anker hat keine Effekte auf den Durchschnitt der als angemessen eingeschätzten maximalen Bezugsdauern. Damit wird auch *H 7* nicht bestätigt. Allerdings verringert der Anker die Varianz der angegebenen Dauern jener Befragten, denen die Rechtslage bekanntgegeben wurde, d. h. die Urteile werden etwas homogener.

Bezieht man zusätzlich Merkmale der Befragten in die Schätzungen mit ein, womit auch mögliche Effekte der leicht verzerrten Stichprobe kontrolliert werden, bleiben die Größenordnungen der Effekte für die Vignettendimensionen sehr ähnlich. Signifikanzniveaus ändern sich in wenigen Fällen (s. Tabelle 6 im Anhang). Bei den Merkmalen der Befragten sind wenige Effekte signifikant – das liegt auch an der relativ geringen Fallzahl und der daraus resultierenden Breite der Konfidenzintervalle. Personen mit einem sehr niedrigen Haushaltseinkommen von maximal 1500 € (+8 Monate im Vergleich zur Referenzkategorie 3000 bis unter 4000 €, *p* < 0,05) sind großzügiger, ebenso Personen mit einem Einkommen von 2000 bis unter 3000 € (+6 Monate, *p* < 0,05). Bei Personen mit geringem Verdienst spricht das Ergebnis für ein Eigeninteresse an sozialstaatlich großzügigeren Leistungen, weil es sich tendenziell eher um Personen mit einer relativ schlechten Arbeitsmarktposition handeln dürfte. Personen, die der Partei DIE LINKE nahestehen, halten im Vergleich zu Anhängerinnen und Anhängern der CDU/CSU längere Bezugsdauern für angemessen (+10 Monate, *p* < 0,05). Befragte, die der Aussage zustimmen, der oder die Einzelne trage keine Schuld an seiner Arbeitslosigkeit, sind ebenfalls großzügiger (+5 Monate, *p* < 0,05). Hingegen sind Personen, die der Aussage zustimmen, man habe es selbst in der Hand, seine berufliche Situation zu ändern, restriktiver (−5 Monate, *p* < 0,01). Personen, die die Wahrscheinlichkeit hoch einschätzen, in den nächsten zwölf Monaten zumindest zeitweise arbeitslos zu sein, plädieren nicht für signifikant längere Bezugsdauern.

## Fazit

Instrumenten und Maßnahmen der Sozialpolitik liegen Gerechtigkeitsvorstellungen in Form von Zuteilungsregeln zugrunde (Leisering 2004; Roosma et al. 2013). Sie spiegeln sich in Demokratien in den in der Bevölkerung vorherrschenden Gerechtigkeitsprinzipien wider, müssen aber mit diesen nicht in jeder Regelung übereinstimmen und können sich mit der Dynamik individueller oder gesellschaftlicher Veränderungen wandeln (Vobruba 1996).

Die maximale Bezugsdauer des Arbeitslosengeldes ist ein Dauerbrenner in wirtschafts- und sozialpolitischen Debatten. Seit Längerem werden von einzelnen Parteien oder Gewerkschaften Ausweitungen der Bezugsdauer und damit eine Rücknahme eines wichtigen Bausteins der Hartz-Reformen gefordert (DIE LINKE 2019; SPD 2019; DGB 2019). Hierbei dient besonders die Berücksichtigung der bisherigen Beschäftigungs- und Beitragsdauer und damit das Leistungs- und Äquivalenzprinzip als Rechtfertigung der Forderungen. Bei den Entscheidungen über Dauern des Arbeitslosengeldbezugs aus der Arbeitslosenversicherung sind Balancen zu finden, zwischen *einerseits* Ansprüchen Arbeitsloser auf Schutz und Neuorientierung und entsprechenden Bedürfnissen sowie Leistungen und Verantwortlichkeiten und *andererseits* einer gesamtwirtschaftlichen Effizienz durch Anreize für schnelle Arbeitsaufnahme oder auch durch Anerkennung bisheriger Leistungen als indirekt produktiv wirkendes Gerechtigkeitsprinzip.

Mit Blick auf Instrumente und Maßnahmen des Gesetzgebers stellt sich die Frage, ob und inwieweit Gerechtigkeitsprinzipien einzelner Regelungen mit denen in der Bevölkerung übereinstimmen und in welcher Weise Gerechtigkeitsprinzipien und ihre Beziehungen zueinander über die Zeit variieren. Variieren sie, etwa in Krisen, dann wäre zu prüfen, ob Instrumente und Maßnahmen der sozialpolitischen Sicherung, die in Deutschland präventiv auch für Krisenfälle hinreichend Wirkung entfallen sollen, dynamischer als bisher auszurichten sind.

Fraglich ist dann unter anderem, ob die maximale Bezugsdauer von Arbeitslosengeld in wirtschaftlichen Krisen steigen sollte und unter welchen Bedingungen dies Menschen befürworten. In den USA gibt es bereits lange regelgebundene eingesetzte „standby extended benefits“ wie auch „emergency benefits“ im Rahmen von Konjunkturpaketen (siehe auch Dietz et al. 2009). Bei regelgebundenen Ausweitungen der Dauern („extended benefits“) sind dabei sowohl das auslösende Ereignis als auch der Umfang und die zeitliche Dauer von Ausweitungen festzulegen. Deutschland hat im Rahmen umfangreicher Sozialschutzpakete die maximale Bezugsdauer in der Covid-19-Pandemie um drei Monate verlängert.

In Krisenzeiten wird die Leistungsfähigkeit sozialpolitischer Sicherungssysteme mit Blick auf Umverteilungen zur Sicherstellung von Teilhabechancen noch einmal in besonderer Weise sichtbar. Zudem verändern sich häufig die Bedingungen der Kriterien, die Verteilungen legitimieren. So beeinflussen wirtschaftliche Krisen beispielsweise die individuelle Kontrolle über die Situation von Arbeitslosen oder die Bedürfnisse von Personen und Haushalten (Konow 1996; Roosma et al. 2016; van Oorschot 2000, S. 36). Dies ist oft auch ein Anlass, sozialpolitisch generösere Regelungen zu fordern.

Vor diesem Hintergrund untersucht die vorliegende Studie, ob Personen in der jüngsten Pandemiekrise in hypothetischen Szenarien und im Vergleich zurzeit vor der Krise für längere Bezugsdauern plädieren und Gerechtigkeitsprinzipien verändert haben.

Die Ergebnisse bestätigen dies nicht. Zumindest im Mai 2020 war die aktuelle Krise für die Befragten offenbar kein Anlass, eine Änderung in den Leistungen der Arbeitslosenversicherung für wünschenswert oder notwendig zu erachten. Die bestehenden Regelungen zur Dauer werden offenbar als effektiv und effizient angesehen. Hierbei kann auch eine Rolle spielen, dass Menschen in Gerechtigkeitserwägungen Wirkungen auf zukünftige Verteilungen berücksichtigen. Wesentlich wäre dann vor allem, ob eine wirtschaftliche Krise als sehr langfristig und die Wirtschaftslage für Arbeitslose als (unverändert) schlecht oder die Situation als kurz- oder mittelfristig vorübergehend angesehen wird (Vobruba 1996).

Die wirtschaftlichen Folgen der Covid-19-Pandemie haben Wirtschaft und Arbeitsmärkte in Ländern unterschiedlich hart getroffen (Eichhorst et al. 2020). In Deutschland konnte dabei Arbeitslosigkeit durch die massive Ausweitung von Kurzarbeit abgefedert werden. Auch dies ist eine mögliche Ursache für fehlende Effekte der Covid-19-Pandemie im Mai 2020 im Vergleich zum November 2019. Gleichwohl waren Grenzschließungen, Lockdown, Lieferkettenunterbrechungen und Produktionsstopps zum Befragungszeitpunkt ein deutliches Bedrohungsszenario mit zugleich erheblicher konjunktureller Wirkungskraft und schnell zeigten sich auch zuvor bestehende Ungleichheit verstärkende Effekte etwa in Form besonderer Risiken für befristet Beschäftigte, Leiharbeitnehmer oder Soloselbstständige, weil letztere oft keinen Schutz durch Kurzarbeit oder die beitragsabhängige Arbeitslosenversicherung hatten (Struck et al. 2021).

Eine wichtige Limitation ist, dass unsere Ergebnisse durch diese Erfahrungen der Menschen in der ersten Hochphase der Covid-19-Pandemie geprägt sind. Inwieweit die Stabilität der Angaben zu Kriterien und Leistungen der Arbeitslosenversicherung vor und während dieser Krise auch auf andere Krisen übertragbar ist, lässt sich nicht abschließend beantworten. Umfassende Krisen sind selten und jede ist in ihren Ursachen und den Bewältigungsversuchen anders. Sie bieten aber wichtige Gelegenheiten für Forschungen etwa zur Wirksamkeit und zur Akzeptanz von Regelungen und Maßnahmen der Sozial- und Arbeitsmarktpolitik, die ja besonders in Krisenzeiten Wirkung zeigen sollen.

Das Paneldesign und der faktorielle Survey ermöglichen es, einzelne (auch durch den Gesetzgeber herangezogene) relevante Merkmale in komplexeren Gesamtszenarien in ihrer spezifischen Bedeutung zu analysieren und dabei die Validität von Aussagen gut zu kontrollieren. Dabei kann der Einfluss dieser Merkmale auf die Beurteilungen vor und während der Covid-19-Krise untersucht werden. Hierbei zeigt sich dann, dass die Befragten der Studie mit dem Alter steigende Bezugsdauern sowohl vor als auch in der Krise unterstützen. Altersabhängige Steigerungen sieht auch die Gesetzeslage vor. Sie tragen den etwas schwierigeren und zum Teil individuell unverschuldeten Wiedereintrittsmöglichkeiten von Personen im höheren Alter Rechnung und berücksichtigen Bedarfs- und Bedürfnissituationen (Kluegel et al. 1999, S. 255; Gilliland 1993; van Oorschot 2000; van Oorschot und Roosma 2017) im Kontext der Verantwortung für die Situation (Konow 1996, 2001). Die Akzeptanz altersabhängiger Bezugsdauern verändert sich in der Krise nicht. Allerdings werden die Differenzen zwischen älteren Arbeitnehmergruppen von den Befragten etwas nivelliert. Während der Gesetzgeber für Personen ab 58 Jahren die maximale Bezugsdauer im Vergleich zu Personen bis 50 Jahren um zwölf Monate erhöht, würden die Befragten älteren Personen eine nur um knapp sechs Monate längere maximale Bezugsdauer zusprechen. Einerseits honorieren Befragte besondere Bedarfslagen krisenunabhängig im höheren Alter, anderseits teilen sie aber auch den jüngeren der hier einbezogenen Altersgruppen etwas längere Bezugsdauern im Vergleich zur Gesetzeslage zu. Befragte berücksichtigen zudem familiäre Sorgearbeit (Pflege des Vaters) und beziehen auch darüber das Gerechtigkeitsprinzip der Bedarfe mit ein (van Oorschot und Roosma 2017). Dies sehen jedoch weder der Gesetzgeber noch von einzelnen Parteien vorgelegte Entwürfe zur Umgestaltung der Dauern in der Arbeitslosenversicherung vor (DIE LINKE 2019; SPD 2019).

In der Politik herrscht vergleichsweise große Einigkeit darüber, die Arbeitslosenversicherung in ihrer Höhe auch weiterhin an den Gerechtigkeitsprinzipien von Äquivalenz und Leistung auszurichten. In den Augen der Befragten soll die Bezugsdauer des Arbeitslosengeldes an vorherige Beitragsleistungen der Arbeitslosen geknüpft und bei entsprechend langjährigen Beitragszahlungen ausgeweitet werden. Hier stehen sich dann direkte und mögliche indirekte gesellschaftliche Effizienz oder Produktivitätswirkungen (Vobruba 1996 sowie Leisering 1999, 2004) gegenüber. Es besteht keine empirische Grundlage für ein Urteil darüber, ob die Wirkungen hinsichtlich einer schnelleren Arbeitsaufnahme bei eher kürzer bemessenen Bezugsdauern, die Wirkungen schlechterer qualifikatorischer und soziale Mismatches am Arbeitsmarkt oder Effekte der Wertschätzung bisheriger Arbeits- und Lebensleitungen insgesamt in der Gesellschaft überwiegen oder ob sie geringer sind. Mangelnde empirische Evidenz bedeutet jedoch nicht, dass an produktivistischer Gerechtigkeit ausgerichtete Argumente der politischen Akteure nicht wichtig dafür sind, dass neue Maßnahmen und Instrumente in der Bevölkerung Akzeptanz finden (Vobruba 1996 sowie Leisering 1999, 2004). „Am Paradigma investiver Sozialpolitik orientierte Reformen“ können offenbar „mit dem Zuspruch der Sozialbürger rechnen“ (Sachweh et al. 2009, S. 618). Aber in unserer Untersuchung hat sich vor allem gezeigt, dass Befragte zugleich und in etwa gleicher Effektstärke sowohl Leistungs- wie auch Bedürftigkeitsmerkmale honorieren.

Insgesamt verdeutlichen die Ergebnisse eine hohe Akzeptanz bestehender Regelungen der maximalen Dauer des Arbeitslosengeldbezugs vor und während der Covid-19-Krise. Die aus Sozialbeiträgen finanzierte Arbeitslosenversicherung und die in ihrer Ausgestaltung festgelegten Gerechtigkeitsprinzipien der Bedürftigkeit (Berücksichtigung erschwerter Austritte höher Altersgruppen aus Arbeitslosigkeit) und von Leistung und Äquivalenz (Berücksichtigung vorheriger Beitragszahlungen) erfahren vor und während der Krise Zustimmung. Die Orientierung an Leistung und Äquivalenz ist jedoch während der Krise geringer als vor der Krise. Im Rahmen der Arbeitslosenversicherung würden die Befragten in gewissem Umfang aber auch sowohl vor und in der Covid-19-Krise eine stärkere Berücksichtigung von Bedürftigkeit bei der Festlegung der maximalen Bezugsdauern als angemessen empfinden. Das Signal an die Mitglieder der Gesellschaft, Lebensleistung wird im Fall unverschuldeter Nichtarbeit vom Sozialstaat honoriert, steht dabei nicht gegen eine gesellschaftlich wirkende investive Sozialpolitik, auch wenn Produktivitätsgewinne schwerer zu berechnen sind als direkte Kosten durch eine Veränderung von Bezugsdauern.

## Anhang

**Table 5:**

Variable	Probit-Modell	
	Koeffizient	Std.-Fehler
Mann (Ref.: Frau)	0,18	0,12
Alter		
Bis 29 Jahre	−0,57*	0,22
30–39 Jahre	−0,40*	0,17
40–49 Jahre	0,00	0,16
50–59 Jahre (Ref.)	–	–
60 Jahre und älter	0,14	0,21
Kinder (Ref.: keine Kinder)	−0,20	0,13
Wohnort Ostdeutschland	0,09	0,17
Haushaltseinkommen		
Unter 1500 €	0,10	0,28
1500 bis unter 2000 €	0,01	0,26
2000 bis unter 3000 €	0,05	0,17
3000 bis unter 4000 € (Ref.)	–	–
4000 bis unter 5000 €	0,15	0,19
5000 € oder mehr	0,44**	0,17
Keine Angabe	0,25	0,23
Parteipräferenz		
CDU/CSU (Ref.)	–	–
SPD	−0,05	0,22
AfD	−0,18	0,36
FDP	0,15	0,32
DIE LINKE	0,29	0,27
Bündnis 90/Die Grünen	0,24	0,18
Andere Partei	0,11	0,35
Keine Partei	0,04	0,23
Unpolitisch	0,42	0,33
Keine Angabe	−0,14	0,20
Zustimmung: „Einzelne/r trägt keine Schuld an Arbeitslosigkeit“	0,20	0,15
Keine Zustimmung: „Einzelne/r trägt keine Schuld an Arbeitslosigkeit“ (Ref.)	–	–
Keine Angabe: „Einzelne/r trägt keine Schuld an Arbeitslosigkeit“	−0,75	0,54
Zustimmung: „Jede/r hat es selber in der Hand, berufliche Situation zu ändern“ (Ref.: keine Zustimmung)	0,01	0,12
Niedrige subjektive Wahrscheinlichkeit, in den nächsten 12 Monaten arbeitslos zu sein	0,09	0,25
Niedrige subjektive Wahrscheinlichkeit, in den nächsten 12 Monaten arbeitslos zu sein (Ref.)		
Keine Angabe: Subjektive Wahrscheinlichkeit, in den nächsten 12 Monaten arbeitslos zu sein	−0,07	0,25
Konstante	−0,62**	0,24
*Pseudo‑R* ^*2*^	*0,05*	
*N (Befragte)*	*592*	

*Quelle*: eigene Berechnungen

Abhängige Variable: Dummy-Variable Teilnahme an Befragung im Mai 2020 (1/0)

*** *p* < 0,001; ** *p* < 0,01; * *p* < 0,05; + *p* < 0,10

**Table 6:**

Unabhängige Variablen	Mittelwert	RE-Modell 3: max. Bezugsdauer mit Personenmerkmalen	
		Koeffizient	Std.-Fehler
**Vignettendimensionen**			
Dummy für Befragung während Corona (Ref.: vor Corona)	0,50	−0,64	0,45
Mann (Ref.: Frau)	0,48	−0,03	0,52
48 Jahre (Ref.)	0,28	–	–
52 Jahre	0,23	1,85**	0,72
56 Jahre	0,25	2,87***	0,67
60 Jahre	0,24	5,73***	0,73
Konkurs des Arbeitgebers (Ref.: Vertrag wegen häufiger Verspätung nicht verlängert)	0,50	3,16***	0,51
In der Vergangenheit durchgängig beschäftigt (Ref.: unregelmäßig beschäftigt)	0,51	4,20***	0,53
Keine Kinder (Ref.)	0,34	–	–
Zwei erwachsene Kinder	0,31	1,08	0,67
Pflegebedürftiger Vater	0,35	2,76***	0,64
Partner/in hat kein Einkommen (Ref.)	0,35	–	–
Partner/in kann Bedarf des Haushalts teilweise decken	0,31	−1,38*	0,63
Partner/in kann Bedarf des Haushalts vollständig decken	0,33	−4,12***	0,62
**Befragtenmerkmale**			
Hinweis zur Rechtslage (Ref.: kein Hinweis)	0,53	−1,65	1,67
Mann (Ref.: Frau)	0,65	2,79	1,78
Alter			
Bis 29 Jahre	0,09	1,81	3,34
30–39 Jahre	0,15	−1,14	2,59
40–49 Jahre (Ref.)	0,23	–	–
50–59 Jahre	0,34	0,74	2,18
60 Jahre und älter	0,19	0,20	3,08
Kinder (Ref.: keine Kinder)	0,62	0,30	1,89
Wohnort Ostdeutschland	0,15	−2,05	2,38
Haushaltseinkommen			
Unter 1500 €	0,05	8,37*	4,11
1500 bis unter 2000 €	0,06	4,16	3,75
2000 bis unter 3000 €	0,17	6,28*	2,85
3000 bis unter 4000 € (Ref.)	0,23	–	–
4000 bis unter 5000 €	0,15	−0,04	2,68
5000 € oder mehr	0,27	−0,74	2,42
Keine Angabe	0,09	2,63	3,40
Parteipräferenz			
CDU/CSU (Ref.)	0,19	–	–
SPD	0,10	1,13	3,20
AfD	0,02	2,19	5,85
FDP	0,04	0,71	4,62
DIE LINKE	0,06	9,88*	3,84
Bündnis 90/Die Grünen	0,29	4,18^+^	2,48
Andere Partei	0,03	4,76	5,28
Keine Partei	0,10	4,32	3,19
Unpolitisch	0,04	4,37	4,54
Keine Angabe	0,13	4,48	2,92
Zustimmung: „Einzelne/r trägt keine Schuld an Arbeitslosigkeit“	0,20	5,07*	2,22
Keine Zustimmung: „Einzelne/r trägt keine Schuld an Arbeitslosigkeit“	0,79	–	–
Keine Angabe: „Einzelne/r trägt keine Schuld an Arbeitslosigkeit“	0,01	−11,54	12,36
Zustimmung: „Jede/r hat es selber in der Hand, berufliche Situation zu ändern“ (Ref.)	0,58	−4,66**	1,77
Keine Zustimmung: „Jede/r hat es selber in der Hand, berufliche Situation zu ändern“	0,42	–	–
Hohe subjektive Wahrscheinlichkeit, in den nächsten 12 Monaten arbeitslos zu sein	0,05	4,66	4,13
Niedrige subjektive Wahrscheinlichkeit, in den nächsten 12 Monaten arbeitslos zu sein (Ref.)	0,85	–	–
Keine Angabe: Wahrscheinlichkeit in den nächsten 12 Monaten arbeitslos zu sein	0,10	−2,64	3,78
Konstante	–	12,88**	3,93
*Pseudo‑R* ^*2*^	*0,25*		
*N (Beobachtungen/Befragte)*	*1488/186*		

Quelle: eigene Berechnungen

*** *p* < 0,001; ** *p* < 0,01; * *p* < 0,05; ^+^ *p* < 0,10

## Footnotes

1 In diese Selektivitätsanalysen wurden 186 Fälle einbezogen, die auch an der Befragung im Mai 2020 teilgenommen hatten und 406 Fälle, die nur im November 2019 teilgenommen hatten, und für die die entsprechenden Analysevariablen vorlagen.

2 Auch ein solches Fehlverhalten kann auf Gründe zurückzuführen sein, über die ein Individuum nicht die alleinige Kontrolle hat, z. B. Krankheit von eigenen Kindern, die Verkehrslage o. Ä. Allerdings sind Beschäftigte in der Pflicht, pünktlich zu sein. Wir unterstellen daher, dass diese Formulierung ein höheres Ausmaß an eigener Kontrolle über die Situation abbildet.

3 Alternativ hätte statt der Angaben aus der Erstbefragung die Antwort „in normalen Zeiten (wie vor der Coronakrise)“ aus der Zweitbefragung herangezogen werden können. Bei dieser Abfrage geben die Befragten eine im Mittel um etwa zwei Monate geringe maximale Bezugsdauer an als in der Erstbefragung. Allerdings handelt es sich dabei um die Ex-post-Bewertung einer kontrafaktischen Situation, die den Befragten mehr Abstraktionsvermögen abverlangt als die Beurteilung der jeweils aktuellen Situation, die wir hier daher als Referenzwert wählen.

4 Der Hausman-Test lehnt die Hypothese gleicher Koeffizienten im Vergleich zu Fixed-Effects-Modellen nicht ab. In diesem Fall ist der Random-Effects-Schätzer effizienter. Die Ergebnisse von FE- und RE-Modellen unterscheiden sich inhaltlich nur in Nuancen.

5 Wir schätzen zudem ein separates Modell, bei dem wir die erste Vignette ausschließen. Die Ergebnisse sind robust.

6 Im Vergleich zur Referenzperson, der 17,2 Monate zugestanden werden, werden ceteris paribus 52-jährigen Arbeitslosen damit in etwa 19,1 Monate Arbeitslosengeld gewährt, 56-jährigen 20,1 Monate und 60-jährigen 23,0 Monate.
